# Epidemiology of Work-Related Injuries Among Construction Workers of Ilam (Western Iran) During 2006 - 2009

**DOI:** 10.5812/ircmj.8011

**Published:** 2013-10-05

**Authors:** Mehdi Moradinazar, Nematullah Kurd, Rozita Farhadi, Vahid Amee, Farid Najafi

**Affiliations:** 1 Imam Khomaini Hospital, Kermanshah University of Medical Sciences, Kermanshah, IR Iran; 2 Kermanshah University of Medical Sciences, Kermanshah, IR Iran; 3 Ilam University of Medical Sciences, Ilam, IR Iran

**Keywords:** Occupational Injury, Incidence, Epidemiology

## Abstract

**Background:**

Work-related injuries are the most important cause of work absence, disability, retirement, mutilation, and even mortality. In Iran a great number of work-related injuries are occurred in construction industry. However, less than 12% of total workers are active in the construction sector.

**Objectives:**

This study aimed to determine the incidence rate of work-related injuries, the type of injuries, and its other determinants among the construction workers of Ilam (Iran).

**Patients and Methods:**

The participants were the workers and staffs working in the construction activities of Ilam in Western Iran. All the recorded injuries and deaths related to the construction workers of Ilam from 2006-2009 were collected from the Bureau of Labor and Social Affairs and then analyzed by the statistical package of SPSS (version 19, for Windows).

**Results:**

During 2006 - 2009 in Ilam, 387workers encountered the building accidents. Their mean age was 34.3 years (SD = 12.4). The average annual incidence of work-related injuries among the workers was 8.2 per 1000 workers. Fracture with 275 cases (71%) was the most common outcome of injuries, and slipping and falling with 77 cases (36%) were the most important events and exposures. The most important factor related to injuries was the lack of surveillance by employers which was also related with the severity of accident-induced injuries (P < 0.004).

**Conclusions:**

Considering the effectiveness of the relevant preventive measures activities such as training the workers as well as using safety tools and more surveillance by employers can decrease the number of work-related injuries among constructive workers.

## 1. Background

Work-related injuries are the most important cause of work absence, disability retirement, mutilation, and even mortality (1, 2). The World Health Organization (WHO) defines the work-related injury as an epidemic problem in the field of public health in developing countries (2, 3). According to article 60 of social security law work-related injuries are some those occurred at work for the worker and inflict some physical and mental injuries. This definition includes those occurred for a worker to save another one (4, 5).

According to the International Labor Organization (ILO), 1 of 10 workers is involved in the injuries annually and 5% of national labor days are lost (6, 7). In the studies conducted in the US, the cost of work-related injuries was 177.2 billion $, and 35 million working days were wasted annually (8-10). According to the reports published by the Social Security Organization (SSO) in 2003, about 150000 occupational injuries were recorded in Iran, of which 1148 (7%) led into death (6, 11, 12). The accidents in any form or degree inflict many economic damages for the worker, employer, and the society. This damage can directly or indirectly affect the individual and society (10, 13-15). Although many attempts are made to reduce occupational morbidities and mortalities (16, 17), such injuries are still one of the most important health problems of developed and developing countries. In fact, due to the lack of international standard registration system for occupational injuries, the existing statistics are not analogues in different countries (13). Based on the statistics published in Iran, the great numbers of work-related injuries are occurred in construction industry and less than 12% of the workers are working in construction sector (13). Unfortunately, regarding the causes of high incidence rate of morbidities and mortalities among construction workers, a few studies have been conducted in Iran (6).

## 2. Objectives

In the present study, we attempted to investigate the incidence and number of such injures, reason and the type of injury among the workers of construction industry in Ilam province. Such data can help in charge authorities to identify the effective factors on the incidence rate of work-related injuries in construction industry.

## 3. Patients and Methods

This was a cross-sectional study and the participants were the workers in the construction activities of Ilam, Western Iran from 2006 to 2009. All the recorded work-related injuries in the construction activities of Ilam were collected from the Bureau of Labor and Social Affairs. After data collection, demographic characteristics including age, gender, and the type of construction injuries, the cause and outcome of the event were extracted. To calculate the incidence of construction injuries, the number of people working in construction sector was extracted from housing and statistics in 2006. The incidence was obtained by dividing the number of cases per year by the number of the workers. For the purpose of this study we used Chi-square tests (for qualitative data) and t-test. Linear regression was used to determine the trend of data. For the analysis of data, the statistical package of SPSS (version 19, windows) was used.

## 4. Results

**Figure 1.**
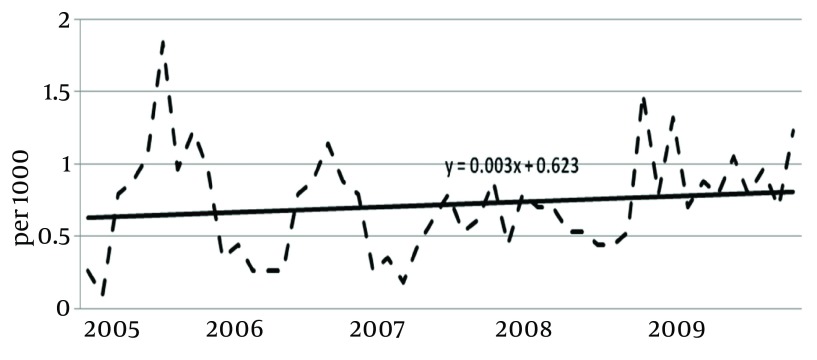
Incidence of Construction Injuries in Ilam During 2006 - 2009

During 2005-2009 in Ilam province, 387 workers of construction sector were injured during construction activities. The mean age was 34.3 ± 12.4 years (range: 15-69 years). The mean of work experience was 6 ± 0.7 years. The mean annual incidence of work-related injuries during the 5-year period of study was 8.2 per 1000 workers. This value for the first and second six-month periods of the years were 10.86 and 6.26 per 1000 worker (P < 0.001). The general trend of occurrence of injuries was ascending during 2006 - 2009 (P = 0.3) (Figure 1). 

**Figure 2.**
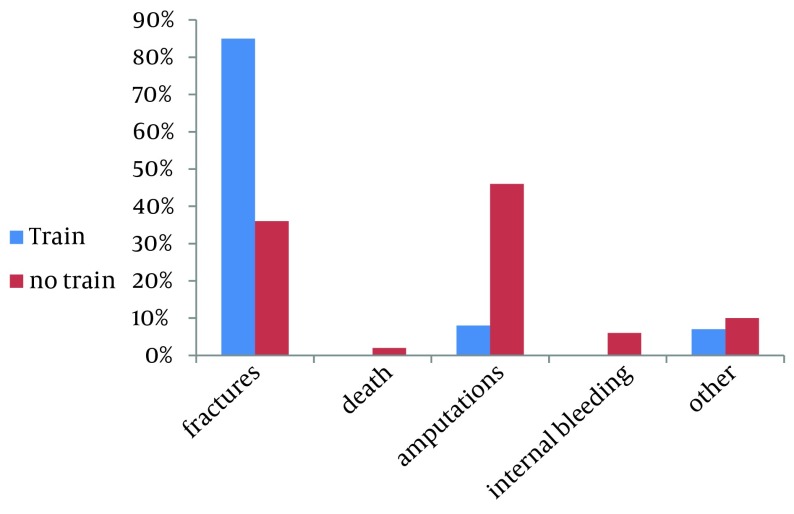
The Severity of Work-Related Injuries Among Construction Workers in Ilam Province by Job Trainings of Labor Office

Further analysis of data showed that there is no significant association between the mean of work experience in year and the severity of the injury (P = 0.25). However, there was an association between job training of labor office and injury severity in construction workers, all 18 deaths and internal bleeding occurred in nontrained people (Figure 2) (P < 0.001). 

**Figure 3.**
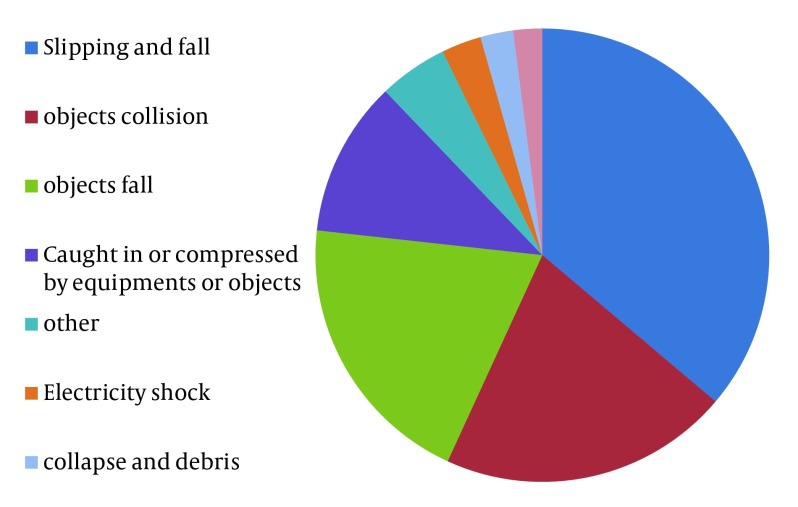
The Events or Exposures Causes Work-Related Injuries Among Construction Workers of Ilam

The most important cause of the work-related injuries in construction workers in Ilam was slip-induced falls which accounted for 77 cases (36%) of total (Figure 3). 

**Figure 4.**
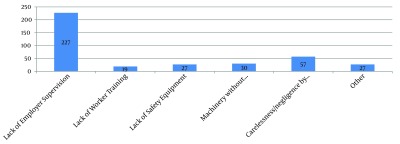
Factors related to Work-Related Injuries Among Construction Workers of Ilam Province

The most important factor related to the construction injuries was the lack of surveillance by the employer (Figure 4). There was a significant association between the events or exposures caused work-related injuries and the type of injuries such that the carelessness of workers and the lack of using protective tools in more than 90% of cases caused fracture. However, the technical defect of machinery and the lack of employers supervision in less than 60% of cases caused fracture. Totally, the lack of supervision caused 80% and 71% of deaths and amputation, respectively (P < 0.004). 

## 5. Discussion

Work-related injuries cause some adverse complications such as fracture, amputation and death by which financial loss and reduction of production are inflicted. Thus, it causes national loss and its prevention is of great importance. To achieve this aim, a multidimensional collaboration should be created between the formal authorities, employers, workers, specialized physicians of labor health, safety committees, and technical safety and other related people and organizations. 

The results of the current study showed that the average annual occurrence of work-related injuries among construction workers of Ilam was 8.2 per thousand which was higher than the results of the similar studies performed in Kerman and Yazd (13, 18). However it was less than values reported in the south-eastern countries of Asia and some of the European countries (19-28). One reason for such differences is related to under reporting of such injuries in Iran as there is no registration system. Factors such as fear of dismiss, interruption of the wages, benefits and insurance premium may cause underreport of real events (29). The average incidence rate of the construction injuries in the first 6 month of study was higher than the second half of the years. This finding was in line with the previous similar studies (13, 18). The higher construction activities and increasing the number of working hours for workers during spring and summer might be the main reason for such differences.

The findings of this study showed that the severity of the occupational accidents in people who received trainings was less than those who did not receive the required courses, and this was in line with the results of the similar studies in Iran and other parts of the world (22, 30-32). Thus, based on the results of this study and other reports regarding the effectiveness of training courses on reducing the severity of the work-related injuries, it seems necessary that social affairs, labor authorities and employers train the workers to reduce occupational injuries among the construction workers which in turn increases their working performance.

The most common end-point of construction injuries was fracture which was in line with the results of other studies in Iran and other countries in which more than 70% of the injuries ended to fracture (22, 30, 31). In addition, our finding was in line with similar reports regarding the falling as one of the main event causing the work-related injuries (22, 25, 30, 31, 33). It is required to routinely use some protective equipment (such as locking hook, rope, scaffold, retaining belt and lanyard) to prevent fall or reduce its consequences. This has been focused in law of working in Islamic Republic of Iran in article 58 where the safety regulations have been obligatory for all workers and workshops by employers.

Furthermore, findings of our study indicated the lack of surveillance by the employer as the most important cause of injuries among construction workers. This finding was in line with the similar studies conducted in Iran (29, 31). Regular visits of inspectors from the Ministry of Health and Bureau of Labor and Social affair can reduce such events. Such inspection has been also focused in law of working in Islamic Republic of Iran in article 98.

Although this study is an epidemiologic study and is faced with its limitations; however, adds to the few reports on work-related injuries in Iran, a developing country with high immigration from rural to urban areas. Immigrates have little training and experiences and therefore enter job opportunities that need little experiences and construction are one of those jobs. That is why the incidence of work-related injuries among developing countries and also among constructive workers is on rise.

Considering the results of the study as well as the importance of construction industries in countries such as Iran, more emphasis on preventive measures such as training the workers and using standard safety tools plus surveillance of the employers can effectively reduce the burden of such injuries. Such preventive strategies are obviously less expensive for workers, employers, and all societies.

## References

[1] Bakhtiyari M, Delpisheh A, Riahi SM, Latifi A, Zayeri F, Salehi M (2012). Epidemiology of occupational accidents among Iranian insured workers.. Safety Sci..

[2] Hämäläinen P (2009). The effect of globalization on occupational accidents.. Safety Sci..

[3] Karvonen M (1986). Epidemiology in the context of occupational health.. Epidemiology of Occupational Health. WHO. M. Karvonen & MI. Mikheev WHO..

[4] Baker SP (1992). The injury fact book..

[5] Williams CA (1991). An international comparison of workers' compensation..

[6] Hämäläinen P, Takala J, Saarela KL (2006). Global estimates of occupational accidents.. Safety Scis..

[7] Servais J-M (2011). International Labour Organization (Ilo)..

[8] Cook TM, Rosecrance J, Zimmermann CL (1996.). Work-related musculoskeletal symptoms among construction workers in the pipe trades..

[9] Dement JM (1999). Workers' compensation experience of North Carolina residential construction workers, 1986-1994.. Appl Occup Environ Hyg..

[10] Larsson TJ, Field B (2002). The distribution of occupational injury risks in the Victorian construction industry.. Safety Sci..

[11] Mehrparvar A, Mirmohammadi S, Ghovve M, Hajian H, Dehghan M, Nabi MR (2011). EPIDEMIOLOGIC STUDY OF OCCUPATIONAL ACCIDENTS RECORDED IN YAZD PROVINCE IN THE YEARS 2007-2008.. Occup Med..

[12] Mohammadfam I, Zokaei HR, Simaei N (2007). Epidemiological evaluation of fatal occupational accidents and estimation of related human costs in Tehran.. Zahedan J Res Med Sci (TABIB-E-SHARGH)..

[13] Bahrampour A, Jafari Nodoushan R, Vatani SJ (2009). Five-year epidemiological study and estimation of accidents distribution in construction industry workers in yazd city by the year 2011 by applying time series model.. J Kerman Univ Med Sci..

[14] Johnson WG, Baldwin ML, Burton Jr JF (1996). Why is the treatment of work-related injuries so costly? New evidence from California.. Inquiry: a journal of medical care organization, provision and financing..

[15] Lowery JT, Glazner J, Borgerding JA, Bondy J, Lezotte DC, Kreiss K (2000). Analysis of construction injury burden by type of work.. Am J Ind Med..

[16] Mohamed S, Ali TH, Tam W (2009). National culture and safe work behaviour of construction workers in Pakistan.. Safety Sci..

[17] Routley V, Valuri J (1994). Work related injuries.

[18] Vatani-Shoaa J, Salasi M, Bahrampour A, Raei M, Asadi M, Jafari-Nodoushan R (2011). An Epidemiological Study of Accidents among Construction Workers in Kerman.. Knowledge & Health..

[19] Dong X, Platner JW (2004). Occupational fatalities of Hispanic construction workers from 1992 to 2000.. Am J Ind Med..

[20] Etiler N, Colak B, Bicer U, Barut N (2004). Fatal occupational injuries among workers in Kocaeli, Turkey, 1990-1999.. Int J Occup Environ Heal..

[21] Jayawardane A, Gunawardena N (1998). Construction workers in developing countries: a case study of Sri Lanka.. Constr Manag Econ..

[22] Jeong BY (1998). Occupational deaths and injuries in the construction industry.. Appl Ergon..

[23] Kisner SM, Fosbroke DE (1994). Injury hazards in the construction industry.. Journal of occupational medicine.: official publication of the Industrial Medical Association..

[24] Koh D, Jeyaratnam J (1998). Occupational health in Singapore.. Int Arch Occ Env hea..

[25] Loomis DP, Richardson DB, Wolf SH, Runyan CW, Butts JD (1997). Fatal occupational injuries in a southern state.. Am J Epidemiol..

[26] Macedo AC, Silva IL (2005). Analysis of occupational accidents in Portugal between 1992 and 2001.. Safety Sci..

[27] Sorock GS, Smith EOH, Goldoft M (1993). Fatal occupational injuries in the New Jersey construction industry, 1983 to 1989.. J Occup Environ Med..

[28] Xia Z, Courtney TK, Sorock GS, Zhu J, Fu H, Liang Y (2000). Fatal occupational injuries in a new development area in the People's Republic of China.. Journal Occup Environ Med..

[29] Isaac D, Pejman B (2011). The incidence and pattern of occupational accidents and related causes in the economic active population Marvdasht city and suburb in 89-84 years.. J Occup Med Spec..

[30] Colak B, Etiler N, Bicer U (2004). Fatal occupational injuries in the construction sector in Kocaeli, Turkey, 1990-2001.. Ind Health..

[31] Halvani G, Ibrahemzadih M (2012). Epidemiological Study and Estimating of Accidents Distribution in Construction Industry Workers in Yazd City by Appling Time Series until 2011.. Int J Occup Saf Heal..

[32] Katsakiori P, Manatakis E, Goutsos S, Athanassiou G (2008). Factors Attributed to Fatal Occupational Accidents in a Period of 5 Years Preceding the Athens 2004 Olympic Games.. Int J Occup Saf Ergon..

[33] Hu K, Rahmandad H, Smith‐Jackson T, Winchester W (2011). Factors influencing the risk of falls in the construction industry: a review of the evidence.. Constr Manag Econ..

